# Grey Rutile TiO_2_ with Long-Term Photocatalytic Activity Synthesized Via Two-Step Calcination

**DOI:** 10.3390/nano10050920

**Published:** 2020-05-09

**Authors:** Yan Liu, Ping Chen, Yaqi Fan, Yanfei Fan, Xifeng Shi, Guanwei Cui, Bo Tang

**Affiliations:** College of Chemistry, Chemical Engineering and Materials Science, Collaborative Innovation Center of Functionalized Probes for Chemical Imaging in Universities of Shandong, Key Laboratory of Molecular and Nano Probes, Ministry of Education, Shandong Normal University, Jinan 250014, China; ly2017020911@163.com (Y.L.); acp1112@163.com (P.C.); fqq93112@163.com (Y.F.); m17865514059@163.com (Y.F.); sxf0716@163.com (X.S.)

**Keywords:** gray color, rutile titanium dioxide, oxygen vacancy, photodecomposition, surface passivation

## Abstract

Colored titanium oxides are usually unstable in the atmosphere. Herein, a gray rutile titanium dioxide is synthesized by two-step calcination successively in a high-temperature reduction atmosphere and in a lower-temperature air atmosphere. The as-synthesized gray rutile TiO_2_ exhibits higher photocatalytic activity than that of white rutile TiO_2_ and shows high chemical stability. This is attributed to interior oxygen vacancies, which can improve the separation and transmission efficiency of the photogenerated carriers. Most notably, a formed surface passivation layer will protect the interior oxygen vacancies and provide long-term photocatalytic activity.

## 1. Introduction

Among titanium oxides, TiO_2_ is well investigated in the photocatalysis research field because of its high chemical stability, low cost, and nontoxicity [1]. However, it can only absorb ultraviolet light, resulting in low photocatalytic efficiency. To expand its light absorbance range and enhance the separation efficiency of the photogenerated carriers, many efforts, such as doping with other elements, sensitizing with dyes, and coupling with metal or nonmetal nanoparticles or different semiconductor materials, have been made to solve the aforementioned problems [2,3,4,5,6,7]. Very recently, TiO_2_ nanotubes synthesized via the electrochemical anodization of titanium foil exhibited visible light response characteristics for the photodecomposition of formaldehyde [8]. 

It has been reported that when TiO_2_ is partially reduced by H_2_ or CO, or bombarded by high-energy particles (laser, electron, or Ar^+^), the obtained colored TiO_2_ powers show visible light photocatalytic activity. In 2010, a blue titanium dioxide with a mixture of anatase and rutile phase was synthesized via hydrolysis and the reduction of isopropyl titanium, showing a higher photocatalytic activity than that of commercial anatase TiO_2_. The higher photocatalytic activity was attributed to the presence of Ti^3+^ in the interior of the titanium dioxide crystal [9]. In 2011, Giamello et al. used an isotope labeling method to study the existence of Ti^3+^ in rutile titanium dioxide in detail [10]. In the same year, a black TiO_2_ with a strong absorption of visible light was synthesized via a high-temperature hydrogenation reduction of P25 TiO_2_ by Chen et al. The obtained higher photoactivity of the black TiO_2_ was attributed to the reduced band gap of titanium dioxide caused by the generation of the surface disordered structure [11]. Another kind of simple method to produce colored TiO_2_ is the addition of fluorine species during TiO_2_ preparation [12,13]. In 2014, Xu et al. synthesized stable blue TiO_2_ nanoparticles with a non-stoichiometric TiO_2−x_ core and stoichiometric TiO_2_ shell structure for the photodecomposition of methylene blue (MB) dyes under visible light irradiation [12]. In addition to the black- or blue-color TiO_2_, Ye et al. found that TiO_2_ nanocrystal assemblies show a yellow color, caused by the interfacial Ti–Ti electronic bonding. Until now, many outstanding works on colored TiO_2_ have been reported [14,15,16,17,18,19,20,21,22,23,24,25,26,27,28]. They usually showed a broader light absorbance range and higher photocatalytic activity than that of white TiO_2_. However, the mechanisms for the higher photocatalytic activity still remain a controversy. Some studies have suggested that it is ascribed to a surface disorder; other reports suggest that it is caused by the “oxygen vacancy” states associated with the Ti^3+^ within the band gap of the TiO_2_ [16,29,30]. Most notably, the reported colored titanium oxides are usually unstable in the atmosphere because of a large number of oxygen vacancies and the presence of Ti^3+^ with poor stability [24]. It is still a challenge to synthesize a stable colored TiO_2_ photocatalyst. 

In contrast to anatase TiO_2_, rutile TiO_2_ has attracted less attention in the photocatalytic research field because of its low photocatalytic activity [31,32]. Herein, gray rutile (GR) titanium dioxide particles (marked as TiO_2_-GR), which are composed of microcrystals with oxygen vacancies on the surface, are synthesized by two-step calcination successively performed in a high-temperature reduction atmosphere and in a lower-temperature air atmosphere. The as-synthesized gray rutile TiO_2_ exhibits a higher photocatalytic activity than that of white rutile (WR) TiO_2_ (marked as TiO_2_-WR). This is attributed to the presence of oxygen vacancies, which can improve the photogenerated carrier separation and transmission efficiency. Most notably, the formed surface passivation layer will protect the interior oxygen vacancies and provide long-term photocatalytic activity.

## 2. Materials and Methods 

### 2.1. Materials

Hexanoic acid (HA), tetrabutyl titanate (TBOT), methylene blue (MB) dye, and glucose were purchased from Sinopharm Chemical Reagent Company. All chemicals were of AR grade. The ultrapure water used in the experiment was obtained from a Mill-Q (electric resistivity 18.2 MΩ·cm) water purification system.

### 2.2. Synthesis of TiO_2_-GR and TiO_2_-WR

**Figure 1:**
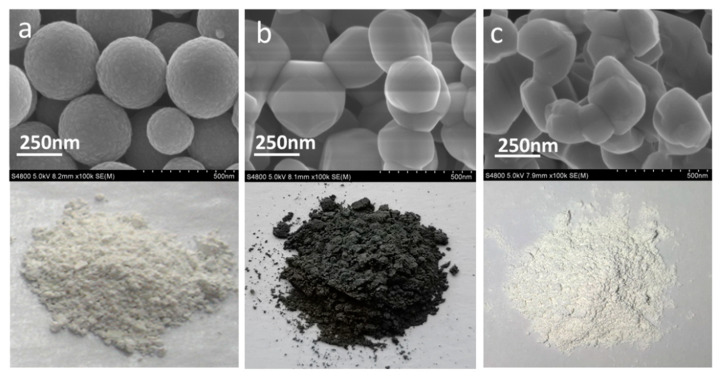
Morphology of spherical anatase TiO_2_ (**a**), TiO_2_-gray rutile (TiO_2_-GR) (**b**), and TiO_2_-white rutile (TiO_2_-WR) (**c**).

First, uniform spherical anatase TiO_2_ particles (Figure 1a and Figure S1a) with a diameter of 200–300 nm were synthesized via a previous reported method [33]. In a typical process, hexanoic acid (0.46 g) dissolved in ethanol (230.0 mL), and TBOT (1.70 g, 10% ethanol solution) was mixed by stirring at room temperature. Then, 35.0 mL H_2_O was dropped into the mixture with vigorous stirring for 12 h at room temperature. The products were obtained after centrifugal separation and were then ready for use for the next two-step calcination procedure. 

Second, the as-prepared TiO_2_ nanosphere was firstly calcinated in a tubular high-temperature furnace with continuous argon flow at 900 °C for 3 h. Then, it was further calcined at 500 °C in air atmosphere for 10 h, and gray rutile TiO_2_ particles with polyhedron morphology were obtained (TiO_2_-GR, Figure 1b and Figure S1b). A white rutile TiO_2_ used as a reference sample (TiO_2_-WR, Figure 1c and Figure S1c) was prepared by the calcination of the as-prepared TiO_2_ nanosphere at 900 °C for 3 h in an air atmosphere. 

The as-prepared photocatalysts were stored in an air atmosphere at room temperature.

### 2.3. Photocurrent Measurements

The photocurrent measurements were carried out on an electrochemical analyzer (CHI660D Instruments, Shanghai Chenhua Instrument Co., Ltd., Shanghai, China) using a standard three-electrode system. The as-prepared samples, a commercial Pt gauze electrode (Gaoss Union Technology Co., Ltd., Wuhan, China, 2 cm × 2 cm, 60 mesh), and saturated calomel electrode were used as working electrodes, counter electrode, and reference electrode, respectively. The working electrode was prepared as follows: 0.05 g of the sample was ground with 0.10 g terpinol for 10 min to make uniform slurry. Then, the slurry was evenly dripped onto a 4.0 cm × 1.0 cm indium tin oxide-coated glass (ITO glass) electrode masked by an adhesive tape with thickness of 0.5 mm and smoothed by a doctor’s blade. Therefore, the formed film about had a thickness of 0.5 mm. Next, these electrodes were dried in an oven and were calcined at 350 °C for 30 min in an air atmosphere. The electrode was immersed in a 0.10 M NaClO_4_ aqueous solution to measure the transient photocurrent under a 300 W Xe arc lamp irradiation with an incident light power density of 130 mW/cm^2^ at 0.4 V vs. the saturated calomel electrode.

### 2.4. Photoactivity Measurements

The photocatalytic discoloration of MB dyes was performed on a reformative XPA-7 photocatalytic reaction instrument(Xujiang Electromechanical Plant, Nanjing, China). The incident light power was 162 mW/cm^2^, which was measured by a handheld Optical Power Meter (Newport 1916-R, Newport Corporation, California, CA, USA). The light exposure area of the quartz bottle was about 19.1 cm^2^. The discoloration effect was measured using the absorption spectroscopic technique. In the typical process, an aqueous solution of the MB dyes (10.0 mg/L and 30.0 mL) and 20.0 mg of the as-prepared photocatalysts were mixed in a 50 mL cylindrical quartz tube and left overnight in darkness to reach the adsorption equilibrium for the MB dyes. Then, the mixture was exposed to 1000 W Xe lamp irradiation with or without the light cutoff filters (*λ* > 420 nm), under ambient conditions and magnetic stirring. At given time intervals, the reaction solution was sampled and analyzed by a UV-visible spectrophotometer (UV 2250, Shimadzu, SHIMADZU (CHINA) Co., Ltd., Shanghai, China). 

## 3. Results and Discussion

A spherical anatase TiO_2_ (Figure 1a and Figure S1a) in a white color was fabricated as the raw material for the gray TiO_2_-GR via the hydrolysis of TBOT in the presence of alkyl chain carboxylic acids [33]. It was determined that alkylchain carboxylic acids remained on the surface of the TiO_2_ nanospheres, which were used as a reductant for the subsequent high-temperature reduction of titanium dioxide [4]. Both the gray rutile TiO_2_ and the reference sample (TiO_2_-WR) exhibited polyhedron morphology (TiO_2_-GR, Figure 1b and Figure S1b). 

**Figure 2:**
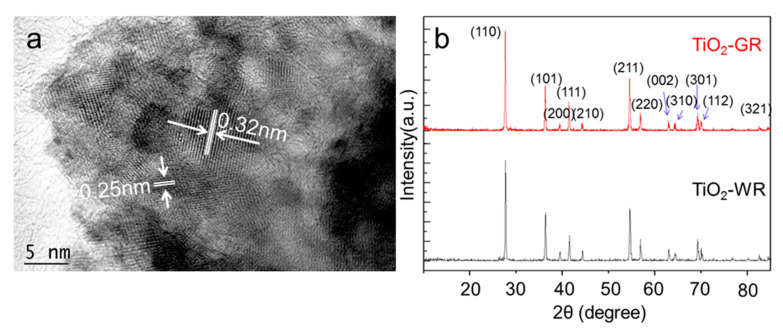
HRTEM (**a**) and XRD patterns (**b**) of gray rutile TiO_2_.

After calcination at 900 °C in an Ar atmosphere, it can be seen from the High Resolution Transmission Electron Microscope (HRTEM) pattern that a surface layer composed of a large number of microcrystals surrounded by a disordered structure formed on the obtained gray TiO_2_ (Figure S2a). The disordered structure is believed to be mainly caused by the presence of oxygen vacancies, which are response for the black color of TiO_2_ [14,30]. Then, after further calcination at 500 °C in an air atmosphere, a dense layer with ordered lattice was formed by the refilling of oxygen atoms into the oxygen vacancies on the outmost layer of TiO_2_ particles (Figure S2b). The formed dense layer with the size of 2–5 nm is on the outermost layer of the TiO_2_-GR particle, which would act as a surface passivation layer to hinder the further diffusion and infiltration of oxygen molecules into the interior oxygen vacancies. As a result, the interior lattice disordered structure would be retained. The lattice width of the surface passivation layer is 0.21 nm, which is ascribed to the (210) crystal faces of the rutile TiO_2_ (JCPDS 21-1276; Figure S2b). However, no such surface layer structures were observed on the surface of the TiO_2_-WR particles (Figure S2c). The lattice widths are 0.32 nm and 0.25 nm, which belong to the (110) and (101) crystal faces of the rutile TiO_2_, respectively (JCPDS 21-1276; Figure 2a). The X-ray diffraction (XRD) peaks of the gray TiO_2_ centered at 2*θ* = 27.75°, 36.4°, 39.45°, 41.55°, 44.35°, 54.6°, 56.9°, 63.05°, 64.35°, 69.25°, 70.05°, and 82.6° are ascribed to the (110), (101), (200), (111), (210), (211), (220), (002), (310), (301), (112), and (321) crystal planes of the rutile TiO_2_, respectively (Figure 2b), which is consistent with the referenced white rutile TiO_2_. Both of the XRD peaks of TiO_2_-GR and TiO_2_-WR are similar to that of the standard rutile TiO_2_ (PDF# 87-0710). The Rietveld analysis (TOPAS V 6.0) of the XRD patterns shows that TiO_2_-GR has an average particle size of 48.5 nm. This result is different from that intuitively observed from the HRTEM patterns, which is attributed to the different detection areas between XRD and HRTEM. Herein, the HRTEM patterns are mainly afforded the surface layer crystal structure of TiO_2_ particles. Therefore, it can be inferred that the as-synthesized TiO_2_-GR nanoparticles are mainly composed of microcrystals with an average size of about 48.5 nm, while their surface layers are composed of smaller microcrystals. Additionally, the analysis results indicate that the broadening of the diffraction peak is mainly due to grain refinement, and there is no existing microstrain. However, compared with the cell parameters of the standard rutile TiO_2_ (PDF# 87-0710), both TiO_2_-GR and TiO_2_-WR show a lattice expansion, with average lattice distortions of 0.11% and 0.13%, respectively. It is proposed that this is mainly caused by the different treatments during the high-temperature calcination process. No peaks centered at 2*θ* = 25.9°, ascribed to carbon (JCPDS 26-1079), were observed in the XRD patterns of gray TiO_2_ [34].

**Figure 3:**
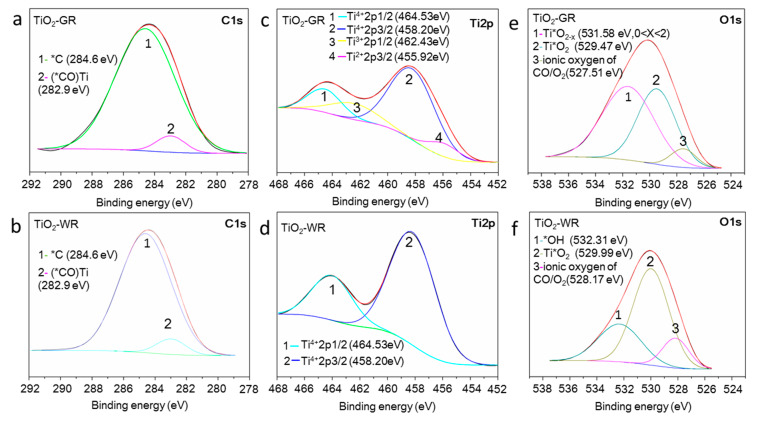
C 1s XPS spectra of TiO_2_-GR (**a**) and TiO_2_-WR (**b**); Ti 2p XPS spectra of TiO_2_-GR (**c**) and TiO_2_-WR (**d**); O 1s XPS spectra of TiO_2_-GR (**e**) and TiO_2_-WR (**f**).

The chemical state of the surface species of TiO_2_-GR and TiO_2_-WR was determined by X-ray photoelectron spectroscopy (XPS), which was further analyzed by an XPS peak-fitting program (version 4.0, Hong Kong, China). The C1s XPS peaks of TiO_2_-GR centered at 284.6 eV (FWHM = 4.55 eV) and 282.9 eV (FWHM = 1.96 eV), which is similar to that of TiO_2_-WR, were ascribed to the *C and (*CO)Ti species caused by the carbon contaminant (Figure 3a,b) [35,36]. It was reported that if a carbon atom was doped in the crystal lattice of TiO_2_, a bonding energy peak ascribed to C* or Ti*–C emerged at 281.6 eV or 454.90 eV, respectively [37,38]. However, no such carbon bonding energy peaks were observed for either TiO_2_-GR or TiO_2_-WR. This indicates that there were no carbon atoms doped in the crystal lattice of the prepared gray TiO_2_, which means that the gray color did not originate from the carbon residues. As shown in Figure 3c,d, the Ti2p XPS peaks of TiO_2_-GR were centered at 464.53 eV (FWHM = 2.84 eV) and 458.20 eV (FWHM = 3.82 eV), which are ascribed to Ti^4+^ 2p_1/2_ and Ti^4+^ 2P_3/2_ of TiO_2_, respectively [39], which is similar to that of TiO_2_-WR. Two reduced titanium ion XPS peaks centered at 462.43 eV (FWHM = 3.91 eV) and 455.92 eV (FWHM = 2.63 eV) were observed for TiO_2_-GR, which could be ascribed to the low valence state titanium of nonstoichiometric TiO_2−x_ (0 < X < 2), mainly including Ti^3+^ 2p_1/2_ of Ti_2_O_3_ and Ti^2+^ 2p_3/2_ of TiO [40,41], which is consistent with the O 1s XPS peak results. However, the TiO_2_-WR showed no reduced titanium ion XPS peaks. The O 1s XPS peaks mainly consisted of three components (Figure 3e,f). The two peaks centered at 529.47 eV (FWHM = 3.30 eV) and 531.58 eV (FWHM = 4.48 eV) were ascribed to the lattice oxygen of the stoichiometric TiO_2_ [42] and nonstoichiometric TiO_2−x_ (0 < X < 2) [43,44], respectively, and the latter may also include some hydroxyl oxygen species [45]. The small O 1s peak centered at 527.51 eV (FWHM = 2.21 eV) could be attributed to the attached ionic oxygen of CO or O_2_ [46]. 

**Figure 4:**
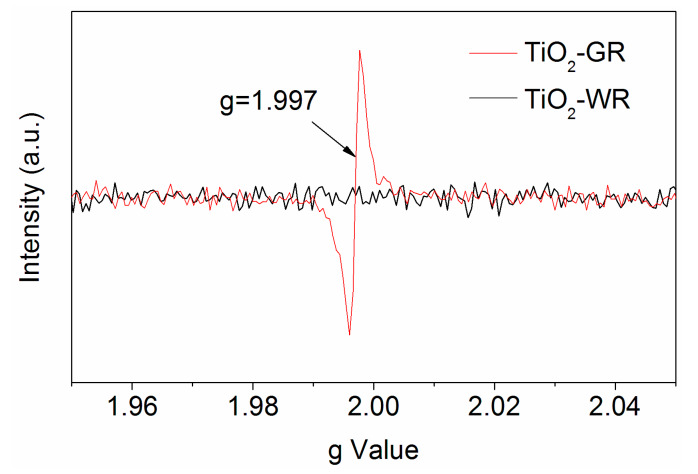
EPR spectroscopy of TiO_2_-GR (red line) and TiO_2_-WR (black line).

It has been reported that the produced Ti^3+^ originated from the oxygen vacancies on the surface of the gray TiO_2_. The removed oxygen atoms left behind two excess electrons per oxygen vacancy, which could be harvested by the neighboring Ti atoms, and induce the formation of Ti^3+^ ions showing EPR signals [47]. Therefore, Electron Paramagnetic Resonance (EPR) is one powerful method for identifying the presence of oxygen vacancies in solid materials. A low-field signal with a g-value close to the free-electron value (*g* = 2.0023) is generally attributed to an unpaired electron trapped on an oxygen vacancy site [11]. Herein, as shown in Figure 4, an EPR signal with a g-value of 1.997 is attributed to the Ti^3+^ centers in the rutile phase environment. As a comparison, there were no EPR peaks at the same position observed from the TiO_2_-WR EPR signals. It is believed that the surface Ti^3+^ would tend to adsorb atmospheric O_2_, which would be reduced to O_2_^−^, and shows an EPR signal at g ≈ 2.02 [11]. The absence of such a peak in the TiO_2_-GR EPR signals indicates that after long calcination in an air atmosphere, the surface oxygen vacancies are refilled by oxygen atoms and the Ti^3+^ is mainly present under the formed surface passivation layer, which is proposed as a key factor for the observed excellent stability of TiO_2_-GR.

**Figure 5:**
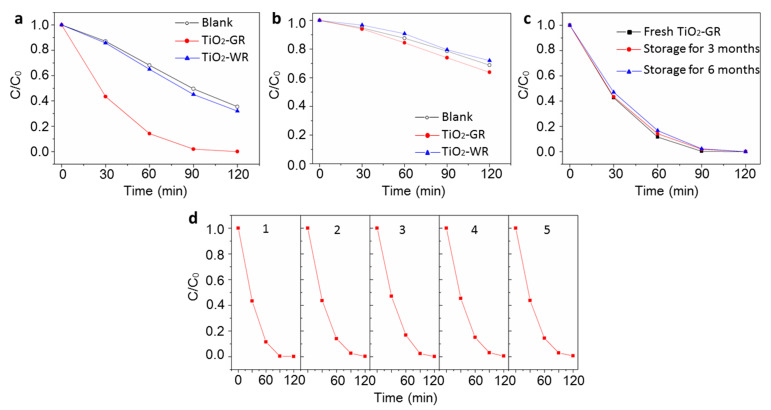
(**a**) Methylene blue (MB) dye photocatalytic discoloration plots of TiO_2_-GR and TiO_2_-WR samples under full-spectrum light irradiation; (**b**) MB dye photocatalytic discoloration plots of TiO_2_-GR and TiO_2_-WR samples under visible light irradiation; (**c**) photocatalytic activity tests of TiO_2_-GR samples stored for different times via the photocatalytic discoloration of MB dyes under full-spectrum light irradiation; (**d**) photocatalytic activity cycle tests of TiO_2_-GR samples under full-spectrum light irradiation. The aqueous solution of the MB dyes (10.0 mg/L, 30.0 mL) without photocatalysts was used as the control sample marked as a blank in Figure 5a,b. C/C_0_ is the ratio of the real-time concentration to the initial concentration of MB dyes.

The photocatalytic activity of the as-prepared TiO_2_-GR was determined by the photocatalytic discoloration of the MB dyes. As shown in Figure 5a,b, the gray TiO_2_ showed a higher photoactivity than that of TiO_2_-WR under visible light or full-spectrum light irradiation. Herein, different from most of the previously reported colored TiO_2_ materials, the as-synthesized gray TiO_2_ showed a long-life photocatalytic activity (Figure 5c,d). The TiO_2_-GR samples retained their color and properties even after six months of storage, and did not exhibit any reduction in their photocatalytic activity after six photocatalysis cycles. This indicates that the gray TiO_2_ has an excellent chemical stability, which is ascribed to the special nanostructure caused by the two-step calcination treatment. As shown in Figure S2b, the formed surface passivation layer will protect the interior oxygen vacancies. As a result, the T^3+^ on the most superficial layer will disappear, which has been confirmed by the aforementioned EPR results. The thermal stability of TiO_2_-GR was further studied by Thermogravimetric Analyzer (TGA) in open air, as shown in Figure S3. The sample was thermally stable up to 650 °C in open air, with negligible weight variation. The slight weight gain and loss wave before 125 °C are ascribed to the adsorption and desorption of O_2_, CO_2_, or H_2_O on the surface of TiO_2_-GR in the air. A distinguishable weight loss from 380 °C is ascribed to the dissociation of the surface –OH. Above 650 °C, the obvious weight increase is ascribed to the refilled interior oxygen vacancies, indicating that the surface passivation layer would be destroyed at this temperature. This shows that the as-prepared TiO_2_-GR has high thermal stability. 

**Figure 6:**
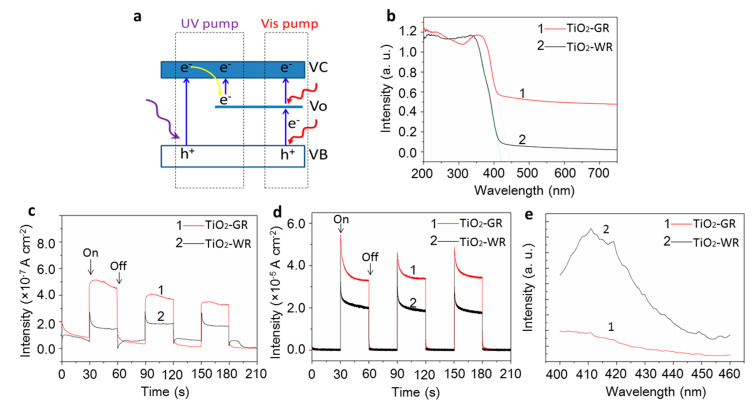
(**a**) Schematic illustration of the photocatalysis mechanism of TiO_2_-GR; (**b**) UV-vis diffuse reflectance spectra of TiO_2_-GR and TiO_2_-WR; (**c**) photocurrent density of TiO_2_-GR and TiO_2_-WR under visible light irradiation; (**d**) photocurrent density of TiO_2_-GR and TiO_2_-WR under full-spectrum light irradiation; (**e**) photoluminescence spectra of TiO_2_-GR and TiO_2_-WR with an excitation wavelength of 380 nm.

A higher photocatalytic activity is attributed to the presence of oxygen vacancies that can create a higher light absorbance and improve the separation and transmission efficiency of photogenerated carriers, which is preliminarily confirmed by the UV-vis spectra, photocurrent, and photoluminescence spectra. The suggested photocatalysis mechanism is shown in Figure 6a. It is proposed that the photocatalysis of TiO_2_-GR may undergo two different photogenerated carrier transfer pathways when pumped by UV light and visible light separately. It can be seen, in both pathways, that the oxygen vacancies all play a vital role. Compared with TiO_2_-WR, the as-prepared TiO_2_-GR exhibits a broad spectral absorption in the visible light region (Figure 6b). This can be attributed to the transitions from the TiO_2_ valence band to the oxygen vacancy levels, or from the oxygen vacancies to the TiO_2_ conduction band pumped by visible light [30], which is responsible for the distinguishable higher photoactivity of TiO_2_-GR than that of TiO_2_-WR. These results are consistent with the photocurrent density results under visible light irradiation (Figure 6c). This indicates that, in this case, the visible light absorption of TiO_2_-GR does lead to charge carrier generation and contributes directly to the photocurrent. However, as shown in Figure 6c,d, the photocurrent density under a full-spectrum light condition is about 100 times that under a visible light condition, which indicates that the contribution of visible light to the improvement of the photocatalytic activity is very limited. This is consistent with previously reported results [48]. Therefore, it is proposed that the main factor for the higher photocatalytic activity of TiO_2_-GR is the photogenerated carrier transfer path pumped by UV light. [49] In this process, the Vo is still proposed to be a key factor for the improvement of the separation efficiency of the photogenerated carriers. First, the Vo can act as a trap site for the temporary storage of electrons, which can be further pumped to the conduction band to react with the substrates, resulting in suppressed recombination of photogenerated carriers. [50]. Herein, the suppressed recombination of photogenerated carriers is preliminarily confirmed by the photoluminescence spectra. If the recombination of carriers was suppressed, the photoluminescence of semiconductor materials would be quenched to some degree [51,52]. As shown in Figure 6e, compared with the reference TiO_2_-WR sample, the TiO_2_-GR samples show a much lower photoluminescence intensity. This indicates that the as-prepared gray TiO_2_-GR has a much higher photoinduced charge separation efficiency than that of the white TiO_2_-GR materials. In addition, because of the presence of free electrons bound loosely to the titanium atom in the oxygen vacancies [47], the surface electric conductivity of TiO_2_-GR will be improved, as a result of improving the carriers’ transmission efficiency [26], which is also helpful for improving the photocatalytic activity. 

## 4. Conclusions

In summary, gray rutile titanium dioxide was synthesized via two-step calcination, performed successively in a high-temperature reduction atmosphere and in a lower-temperature air atmosphere. The results indicate that, compared with the white rutile titanium dioxide, the as-prepared gray titanium dioxide exhibits the typical characteristics of black- or blue-color TiO_2_, such as the presence of Ti ions in a low valence state, surface disorder structure, and oxygen vacancies, which are caused by the loss of oxygen atoms under reduction reaction conditions. According to previous reports [14], it is proposed that the presence of Ti^3+^ or a surface disorder structure is mainly induced by oxygen vacancies. The as-synthesized gray titanium dioxide exhibits a higher photocatalytic activity than does white rutile TiO_2_. This is attributed to the interior vacancies, which can create a higher light absorbance and improve the separation and transmission efficiency of photogenerated carriers. Most notably, it is proposed that the two-step calcination can produce a surface passivation layer on the surface of gray titanium dioxide particles, as a result of protecting the interior oxygen vacancies, which provides long-term photocatalytic activity. This study provides a considerable reference for the design and synthesis of other semiconductor photocatalysts rich in oxygen vacancies, with high activity and high stability.

## Supplementary Materials

The following are available online at https://www.mdpi.com/2079-4991/10/5/920/s1: Figure S1: Morphology of spherical anatase TiO_2_ (a), TiO_2_-GR (b), and TiO_2_-WR (c). Figure S2: HRTEM of TiO_2_-only calcination at 900 °C in Ar atmosphere (a), TiO_2_-GR (b), and TiO_2_-WR (c). Figure S3: TGA curve in open air for the TiO_2_-GR sample.

## References

[1] Chen X., Mao S.S. (2007). Titanium Dioxide Nanomaterials: Synthesis, Properties, Modifications, and Applications. Chem. Rev..

[2] Kubacka A., Fernández-García M., Colón G. (2012). Advanced Nanoarchitectures for Solar Photocatalytic Applications. Chem. Rev..

[3] Xie M., Fu X., Jing L., Luan P., Feng Y., Fu H. (2014). Long-Lived, Visible-Light-Excited Charge Carriers of TiO_2_/BiVO_4_ Nanocomposites and their Unexpected Photoactivity for Water Splitting. Adv. Energy Mater..

[4] Cui G., Wang W., Ma M., Zhang M., Xia X., Han F., Shi X., Zhao Y., Dong Y., Tang B. (2013). Rational design of carbon and TiO_2_ assembly materials: Covered or strewn, which is better for photocatalysis?. Chem. Commun..

[5] Linh V.T.N., Xiao X., Jung H.S., Giannini V., Maier S.A., Kim D.-H., Lee Y.-I., Park S.-G. (2019). Compact Integration of TiO_2_ Nanoparticles into the Cross-Points of 3D Vertically Stacked Ag Nanowires for Plasmon-Enhanced Photocatalysis. Nanomaterials.

[6] Zhang Z., Liu H., Wang X., Zhang J., Yu M., Ramakrishna S., Long Y. (2019). One-Step Low Temperature Hydrothermal Synthesis of Flexible TiO_2_/PVDF@MoS_2_ Core-Shell Heterostructured Fibers for Visible-Light-Driven Photocatalysis and Self-Cleaning. Nanomaterials.

[7] Jiang X., Xu W., Yu L. (2019). Photocatalytic Decomposition of Gaseous HCHO over Ag Modified TiO_2_ Nanosheets at Ambient Temperature. Nanomaterials.

[8] Sahrin N.T., Nawaz R., Kait C.F., Lee S.L., Wirzal M.D.H. (2020). Visible Light Photodegradation of Formaldehyde over TiO_2_ Nanotubes Synthesized via Electrochemical Anodization of Titanium Foil. Nanomaterials.

[9] Zuo F., Wang L., Wu T., Zhang Z., Borchardt D., Feng P. (2010). Self-Doped Ti^3+^ Enhanced Photocatalyst for Hydrogen Production under Visible Light. J. Am. Chem. Soc..

[10] Livraghi S., Maurelli S., Paganini M.C., Chiesa M., Giamello E. (2011). Probing the Local Environment of Ti^3+^ Ions in TiO_2_ (Rutile) by ^17^O HYSCORE. Angew. Chem. Int. Ed..

[11] Chen X., Liu L., Yu P.Y., Mao S.S. (2011). Increasing Solar Absorption for Photocatalysis with Black Hydrogenated Titanium Dioxide Nanocrystals. Science.

[12] Zhu Q., Peng Y., Lin L., Fan C.-M., Gao G.-Q., Wang R.-X., Xu A.-W. (2014). Stable blue TiO_2−x_ nanoparticles for efficient visible light photocatalysts. J. Mater. Chem. A.

[13] Bellardita M., Garlisi C., Ozer L.Y., Venezia A.M., Sá J., Mamedov F., Palmisano L., Palmisano G. (2020). Highly stable defective TiO_2−x_ with tuned exposed facets induced by fluorine: Impact of surface and bulk properties on selective UV/visible alcohol photo-oxidation. Appl. Surf. Sci..

[14] Naldoni A., Allieta M., Santangelo S., Marelli M., Fabbri F., Cappelli S., Bianchi C.L., Psaro R., Santo V.D. (2012). The effect of nature and location of defects on bandgap narrowing in black TiO_2_ nanoparticles. J. Am. Chem. Soc..

[15] Hamdy M.S., Amrollahi R., Mul G. (2012). Surface Ti^3+^-Containing (blue) Titania: A Unique Photocatalyst with High Activity and Selectivity in Visible Light-Stimulated Selective Oxidation. ACS Catal..

[16] Hu Y.H. (2012). A Highly Efficient Photocatalyst-Hydrogenated Black TiO_2_ for the Photocatalytic Splitting of Water. Angew. Chem. Int. Ed..

[17] Grabstanowicz L.R., Gao S., Li T., Rickard R.M., Rajh T., Liu D.-J., Xu T. (2013). Facile Oxidative Conversion of TiH_2_ to High-Concentration Ti^3+^-Self-Doped Rutile TiO_2_ with Visible-Light Photoactivity. Inorg. Chem..

[18] Zhou W., Li W., Wang J., Qu Y., Yang Y., Xie Y., Zhang K., Wang L., Fu H., Zhao D. (2014). Ordered Mesoporous Black TiO_2_ as Highly Efficient Hydrogen Evolution Photocatalyst. J. Am. Chem. Soc..

[19] Liu N., Schneider C., Freitag D., Hartmann M., Venkatesan U., Müller J., Spiecker E., Schmuki P. (2014). Black TiO_2_ Nanotubes: Cocatalyst-Free Open-Circuit Hydrogen Generation. Nano Lett..

[20] Zhao Z., Zhang X., Zhang G., Liu Z., Qu D., Miao X., Feng P., Sun Z. (2015). Effect of defects on photocatalytic activity of rutile TiO_2_ nanorods. Nano Res..

[21] Liu N., Haublein V., Zhou X., Venkatesan U., Hartmann M., Mackovic M., Nakajima T., Spiecker E., Osvet A., Frey L. (2015). “Black” TiO_2_ Nanotubes Formed by High-Energy Proton Implantation Show Noble-Metal-co-Catalyst Free Photocatalytic H_2_-Evolution. Nano Lett..

[22] Chen J., Song W., Hou H., Zhang Y., Jing M., Jia X., Ji X. (2015). Ti^3+^ Self-Doped Dark Rutile TiO_2_ Ultrafine Nanorods with Durable High-Rate Capability for Lithium-Ion Batteries. Adv. Funct. Mater..

[23] Tian M., Mahjouri-Samani M., Eres G., Sachan R., Yoon M., Chisholm M.F., Wang K., Puretzky A.A., Rouleau C.M., Geohegan D.B. (2015). Structure and Formation Mechanism of Black TiO_2_ Nanoparticles. ACS Nano.

[24] Chen S., Tao J., Tao H., Wang C., Shen Y., Jiang J., Zhu L., Zeng X., Wang T. (2016). One-Step Solvothermal Synthesis of Black TiO_2_ Films for Enhanced Visible Absorption. J. Nanosci. Nanotechnol..

[25] Chen X., Zhao D., Liu K., Wang C., Liu L., Li B., Zhang Z., Shen D. (2015). Laser-Modified Black Titanium Oxide Nanospheres and Their Photocatalytic Activities under Visible Light. ACS Appl. Mater. Interfaces.

[26] Lv X., Chen A., Luo Y., Lu P., Dai Y., Enriquez E., Dowden P., Xu H., Kotula P.G., Azad A.K. (2016). Conducting Interface in Oxide Homojunction: Understanding of Superior Properties in Black TiO_2_. Nano Lett..

[27] Zhou Y., Chen C., Wang N., Li Y., Ding H. (2016). Stable Ti^3+^ Self-Doped Anatase-Rutile Mixed TiO_2_ with Enhanced Visible Light Utilization and Durability. J. Phys. Chem. C.

[28] Chen S., Xiao Y., Wang Y., Hu Z., Zhao H., Xie W. (2018). A Facile Approach to Prepare Black TiO_2_ with Oxygen Vacancy for Enhancing Photocatalytic Activity. Nanomaterials.

[29] Chen X., Liu L., Liu Z., Marcus M.A., Wang W., Oyler N.A., Grass M.E., Mao B., Glans P.-A., Yu P.Y. (2013). Properties of Disorder-Engineered Black Titanium Dioxide Nanoparticles through Hydrogenation. Sci. Rep..

[30] Yu P., Zhang J. (2015). Some interesting properties of black hydrogen-treated TiO_2_ nanowires and their potential application in solar energy conversion. Sci. China Chem..

[31] Li X., Xiong Y., Li Z., Xie Y. (2006). Large-Scale Fabrication of TiO_2_ Hierarchical Hollow Spheres. Inorg. Chem..

[32] Andersson M., Osterlund L., Ljungstrom S., Palmqvist A. (2002). Preparation of Nanosize Anatase and Rutile TiO_2_ by Hydrothermal Treatment of Microemulsions and Their Activity for Photocatalytic Wet Oxidation of Phenol. J. Phys. Chem. B.

[33] Liu S., Han G., Shu M., Han L., Che S. (2010). Monodispersed inorganic/organic hybrid spherical colloids: Versatile synthesis and their gas-triggered reversibly switchable wettability. J. Mater. Chem..

[34] Li Y., Zhu S., Liu Q., Gu J., Guo Z., Chen Z., Feng C., Zhang D., Moon W.-J. (2012). Carbon-coated SnO_2_@C with hierarchically porous structures and graphite layers inside for a high-performance lithium-ion battery. J. Mater. Chem..

[35] Xie Y., Sherwood P.M.A. (1992). Ultrahigh Purity Graphite Electrode by Core Level and Valence Band XPS. Surf. Sci. Spectra.

[36] Ocal C., Ferrer S. (1986). The strong metal–support interaction (SMSI) in Pt–TiO_2_ model catalysts. A new CO adsorption state on Pt–Ti atoms. J. Chem. Phys..

[37] Liu G., Han C., Pelaez M., Zhu D., Liao S., Likodimos V., Ioannidis N., Kontos A.G., Falaras P., Dunlop P.S.M. (2012). Synthesis, characterization and photocatalytic evaluation of visible light activated C-doped TiO_2_ nanoparticles. Nanotechnology.

[38] Galuska A.A., Uht J.C., Marquez N. (1988). Reactive and nonreactive ion mixing of Ti films on carbon substrates. J. Vac. Sci. Technol. A.

[39] Dementjev A.P., Ivanova O.P., Vasilyev L.A., Naumkin A.V., Nemirovsky D.M., Shalaev D.Y. (1994). Altered layer as sensitive initial chemical state indicator. J. Vac. Sci. Technol. A.

[40] Gonbeau D., Guimon C., Pfister-Guillouzo G., Levasseur A., Meunier G., Dormoy R. (1991). XPS study of thin films of titanium oxysulfides. Surf. Sci..

[41] Franzen H.F., Umana M.X., McCreary J.R., Thorn R.J. (1976). XPS spectra of some transition metal and alkaline earth monochalcogenides. J. Solid State Chem..

[42] Haukka S., Lakomaa E.-L., Jylha O., Vilhunen J., Hornytzkyj S. (1993). Dispersion and distribution of titanium species bound to silica from titanium tetrachloride. Langmuir.

[43] Kuznetsov M.V., Zhuravlev J.F., Gubanov V.A. (1992). XPS analysis of adsorption of oxygen molecules on the surface of Ti and TiNx films in vacuum. J. Electron. Spectrosc. Relat. Phenom..

[44] Kuznetsov M.V., Zhuravlev J.F., Zhilyaev V.A., Gubanov V.A. (1992). XPS study of the nitrides, oxides and oxynitrides of titanium. J. Electron. Spectrosc. Relat. Phenom..

[45] Tan B.J., Klabunde K.J., Sherwood P.M.A. (1990). X-ray photoelectron spectroscopy studies of solvated metal atom dispersed catalysts. Monometallic iron and bimetallic iron-cobalt particles on alumina. Chem. Mater..

[46] Bukhtiyarov V.I., Boronin A.I., Prosvirin I.P., Savchenko V.I.J. (1994). Stages in the Modification of a Silver Surface for Catalysis of the Partial Oxidation of Ethylene: II. Action of the Reaction Medium. J. Catal..

[47] Deskins N.A., Rousseau R., Dupuis M. (2011). Distribution of Ti^3+^ Surface Sites in Reduced TiO_2_. J. Phys. Chem. C.

[48] Wang G., Wang H., Ling Y., Tang Y., Yang X., Fitzmorris R.C., Wang C., Zhang J.Z., Li Y. (2011). Hydrogen-treated TiO_2_ nanowire arrays for photoelectrochemical water splitting. Nano Lett..

[49] Wheeler D.A., Ling Y.C., Dillon R.J., Fitzmorris R.C., Dudzik C.G., Zavodivker L., Rajh T., Dimitrijevic N.M., Millhauser G., Bardeen C. (2013). Probing the nature of bandgap states in hydrogen-treated TiO_2_ nanowires. J. Phys. Chem..

[50] Wheeler D.A., Wang G., Fitzmorris R.C., Adams S.A., Li Y., Zhang J.Z. (2012). Ultrafast charge carrier dynamics and photoelectrochemical properties of hydrogen-treated TiO_2_ nanowire arrays. MRS Proc..

[51] Li H., Liu G., Chen S., Liu Q. (2010). Novel Fe doped mesoporous TiO_2_ microspheres: Ultrasonic–hydrothermal synthesis, characterization, and photocatalytic properties. Physica E.

[52] Acharya K.P., Khnayzer R.S., Connor T., Diederich G., Kirsanova M., Klinkova A., Roth D., Kinder E., Imboden M., Zamkov M. (2011). The Role of Hole Localization in Sacrificial Hydrogen Production by Semiconductor–Metal Heterostructured Nanocrystals. Nano Lett..

