# Supplementary material for: Grey Rutile TiO2 with Long-Term Photocatalytic Activity Synthesized Via Two-Step Calcination
Source: Nanomaterials (Basel). 2020 May 9;10(5):920. doi: 10.3390/nano10050920 (PMC7279311; doi:10.3390/nano10050920)
Supplement: Supplementary file 1 [file nanomaterials-10-00920-s001.pdf]

Supplementary Material

Grey Rutile TiO_2 with Long-Term Photocatalytic Activity Synthesized Via Two-Step Calcination

Yan Liu, Ping Chen, Yaqi Fan, Yanfei Fan, Xifeng Shi, Guanwei Cui * and Bo Tang *

College of Chemistry, Chemical Engineering and Materials Science, Collaborative Innovation Center of Functionalized Probes for Chemical Imaging in Universities of Shandong, Key Laboratory of Molecular and Nano Probes, Ministry of Education, Shandong Normal University, Jinan 250014, China; ly2017020911@163.com (Y.L.); acp1112@163.com (P.C.); fqq93112@163.com (Y.Q.F.); m17865514059@163.com (Y.F.F.); sxf0716@163.com (X.S.)

* Correspondence: cuiguanwei@sdnu.edu.cn (G.C.), tangb@sdnu.edu.cn (B.T.); Tel.: +86-1358-906-3951 (G.C.)

1. Physical Characterization

A HITACHI S-4800 Scanning Electron Microscope (SEM) equipped with a field-emission gun operated at 5.0 kV was used to characterize the morphology of the as-obtained product. Transmission electron microscopic (TEM) images, high-resolution transmission electron microscopic (HRTEM) images were obtained using a TECNAI G2 high resolution transmission electron microscope operating at 200 kV. The crystalline structures of the samples were evaluated by X-ray diffraction (XRD) analyses carried out on a Bruker D8 Advance Diffractmeter with Cu Kα radiation (1.5418 Å). X-ray photoelectron spectroscopy (XPS) spectra were performed with a Phobios 100 electron analyzer (SPECS GmbH) equipped 5 channeltrons, using an unmonochromated Mg Kα X-ray source (1253.6 eV). The UV-vis diffuse reflectance spectrum was measured on a UV-2550 SHIMADZ spectrophotometer with $BaSO_4$ powder used as a reference (100% reflectance). Electron paramagnetic resonance (EPR) signals were recorded on a Bruker A300-10/12/S-LC spectrometer with a frequency of 9.87 GHz at low temperature. The Photoluminescence spectrum was carried out with an Edinburgh FLS920 spectrofluorimeter (Edinburgh Instruments Ltd, Livingston, England) equipped with a xenon lamp. Thermogravimetric Analysis (TGA) was performed on METTLER TOLEDO TGD/DSC 3+ with a heating rate of 5 °C min^{-1}.

2. Supplementary Figures

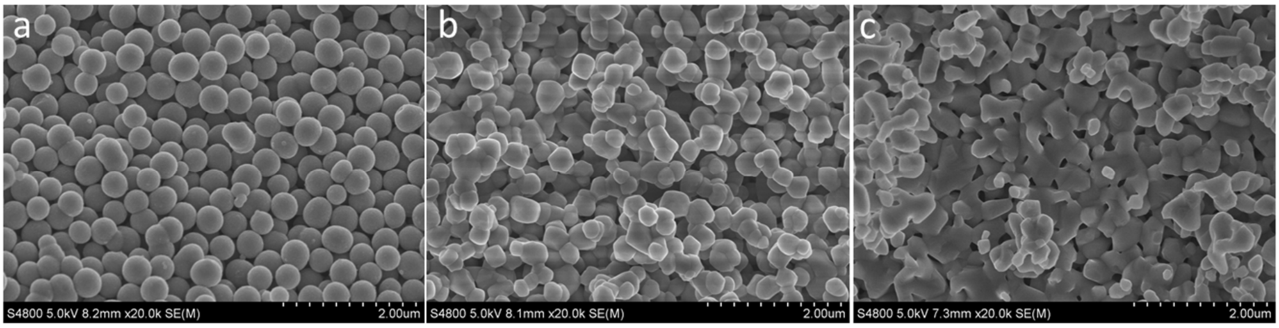

Figure S1. Morphology of spherical anatase TiO_2 (**a**), TiO_2-GR (**b**) and TiO_2-WR (**c**).

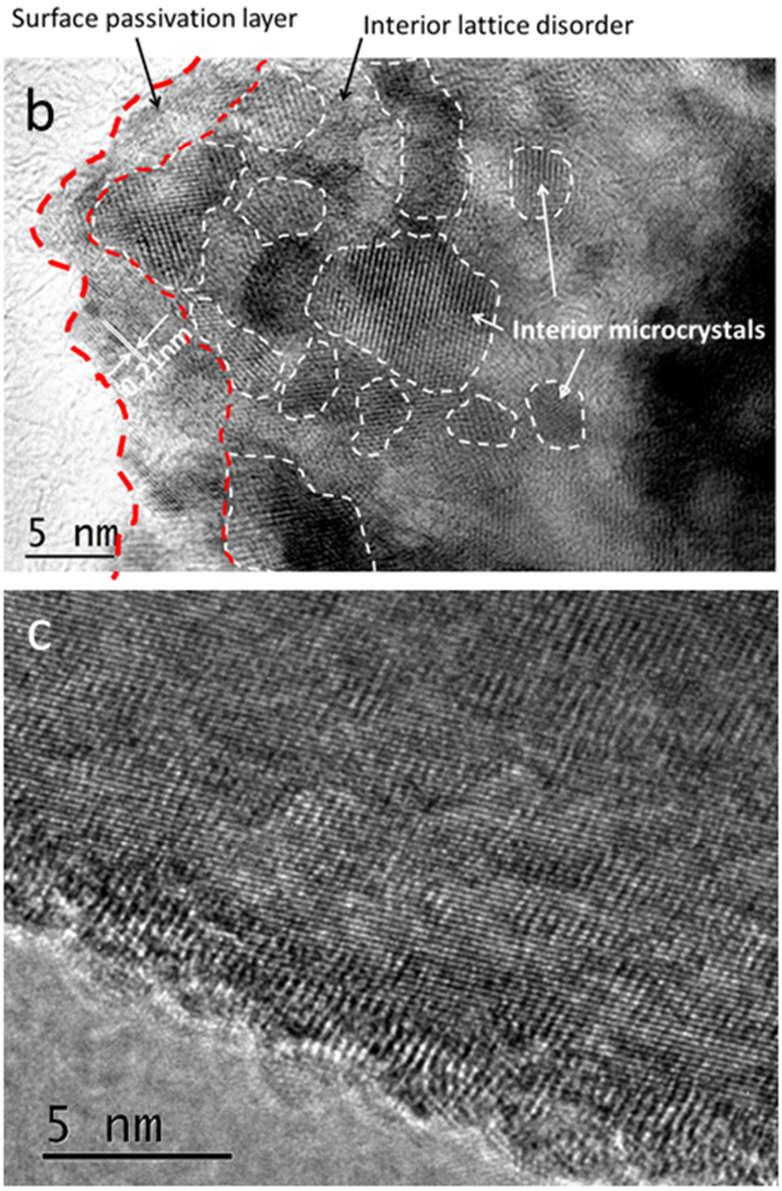

Figure S2. HRTEM of TiO_2 only calcination at 900 °C in Ar atmosphere (**a**), TiO_2-GR (**b**) and TiO_2-WR (**c**).

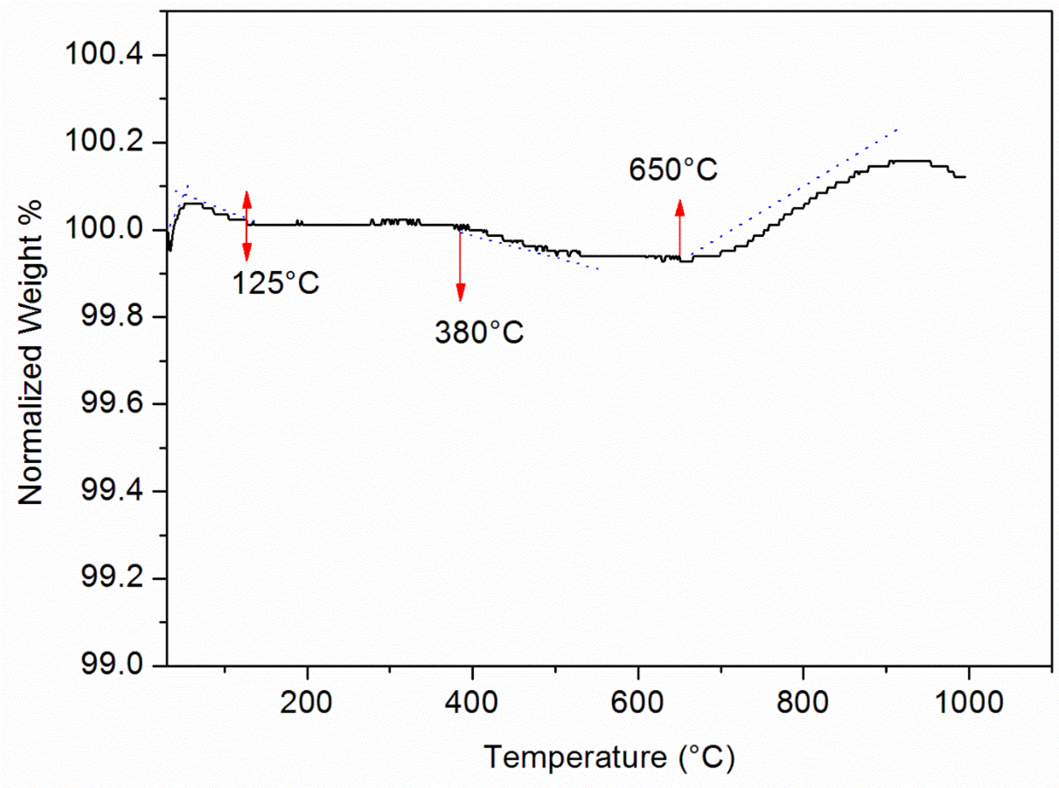

Figure S3. TGA curve in open air for TiO_2-GR sample.
